# Peek Community Eye Health - mHealth system to increase access and efficiency of eye health services in Trans Nzoia County, Kenya: study protocol for a cluster randomised controlled trial

**DOI:** 10.1186/s13063-019-3615-x

**Published:** 2019-08-14

**Authors:** Hillary Rono, Andrew Bastawrous, David Macleod, Emmanuel Wanjala, Stephen Gichuhi, Matthew Burton

**Affiliations:** 1 International Centre for Eye Health, Clinical Research Department, Faculty of Infectious and Tropical Diseases, London School of Hygiene & Tropical Medicine, Keppel Street, London, WC1E 7HT UK; 2 Kitale County referral and teaching Hospital, Ravine Road, P.O. Box 98, Kitale, 30200 Kenya; 3 Department of Ophthalmology, University of Nairobi, P.O. Box 19676, Nairobi, 00202 Kenya; 4 The Peek Vision Foundation, 1 Fore Street, London, EC2Y 9DT UK

**Keywords:** Eye problems, Visual impairement, Access, Primary eye care, Community Eye Health system, Community volunteers, Peek community screening App, Cluster randomised controlled trial

## Abstract

**Background:**

Globally, eye care provision is currently insufficient to meet the requirement for eye care services. Lack of access and awareness are key barriers to specialist services; in addition, specialist services are over-utilised by people with conditions that could be managed in the community or primary care. In combination, these lead to a large unmet need for eye health provision.

We have developed a validated smartphone-based screening algorithm (Peek Community Screening App). The application (App) is part of the Peek Community Eye Health system (Peek CEH) that enables Community Volunteers (CV) to make referral decisions about patients with eye problems. It generates referrals, automated short messages service (SMS) notifications to patients or guardians and has a program dashboard for visualising service delivery.

We hypothesise that a greater proportion of people with eye problems will be identified using the Peek CEH system and that there will be increased uptake of referrals, compared to those identified and referred using the current community screening approaches.

**Study design:**

A single masked, cluster randomised controlled trial design will be used. The unit of randomisation will be the ‘community unit’, defined as a dispensary or health centre with its catchment population. The community units will be allocated to receive either the intervention (Peek CEH system) or the current care (periodic health centre-based outreach clinics with onward referral for further treatment). In both arms, a triage clinic will be held at the link health facility four weeks from sensitisation, where attendance will be ascertained. During triage, participants will be assessed and treated and, if necessary, referred onwards to Kitale Eye Unit.

**Discussion:**

We aim to evaluate a M-health system (Peek CEH) geared towards reducing avoidable blindness through early identification and improved adherence to referral for those with eye problems and reducing demand at secondary care for conditions that can be managed effectively at primary care level.

**Trial registration:**

The Pan African Clinical Trials Registry (PACTR), 201807329096632. Registered on 8 June 2018.

**Electronic supplementary material:**

The online version of this article (10.1186/s13063-019-3615-x) contains supplementary material, which is available to authorized users.

## Background

Globally, it is estimated that 253 million people have visual impairment (VI; visual acuity in the better eye < 6/18), 36 million of whom are blind (visual acuity in the better eye < 3/60) [1]. About 80% of the impairment is avoidable [2]. Approximately 90% of those who are living with VI are in low- and middle-income countries [3]. Although the prevalence of moderate or severe vision impairment in adults aged ≥ 50 years is higher in South and Southeast Asia, North Africa, and the Middle East [4], sub-Saharan Africa (SSA) has the greatest gap between need (blindness VI) and available eye services [5]. In Kenya, the prevalence of blindness is high; it is in the range of 0.6–2.0%, depending on the region [6–10]. There are only 115 ophthalmologists for a population of 49 million. Moreover, their distribution is very uneven, in the range of 0–17 per 1 million population across the various counties [11].

The causes of blindness vary according to regions and countries [12–15]. Globally, the leading causes of VI are uncorrected refractive error and cataract, while cataract and glaucoma are the leading causes of blindness [2, 16]. Other causes of blindness include diabetes, macular degeneration, and other posterior eye diseases [7, 10, 17].

The reasons for a high burden of VI include poverty and a lack of access to eye services [18]. Patient factors such as lack of awareness, fear of treatment outcomes, increasing age, female gender, and presence of diabetes increase the risk of blindness [10, 19]. Health system-related factors include low numbers of eye workers, variable productivity, high indirect and direct costs, and the mal-distribution of the work force, which currently favours major urban areas [20–23]. In addition, there are ‘provider’ factors, such as poor-quality services arising from a shortage of trained staff and infrastructure [19, 24]. There is a large disparity between the need for eye services and availability of eye care workers [5].

To improve access to eye health services, especially in rural areas, outreach programs designed to promote access to eye services by communities in remote regions have been used [22, 25]. They provide short-term access to eye services for patients; however, the long-term goal is to integrate eye services into primary healthcare (PHC) as a continuum of health service provision [26, 27]. Redistribution of tasks among health workforce teams, to improve efficiency among available human resources, have also been used with variable success [9, 28]. Effective task shifting with clear referral criteria and management plans has been successfully delivered through algorithms such as the Integrated Management of Childhood Illness (IMCI) at primary level [29, 30]. In eye care, decision trees and algorithms have been developed, mostly outside Africa, and focused on identifying the diagnosis and treatment at a secondary level [31–33]. The World Health Organization (WHO) recently developed similar algorithms and training manual for use at the PHC facilities in Africa [34]. To our knowledge, there are no digital algorithms to identify and refer people from communities.

### Rationale

There is a clear need for improved access to eye health services for populations in many regions of the world. Availability of mobile phone technology and its usage in healthcare, including eye care, is increasing rapidly [35, 36]. One such example is Peek acuity, which has developed applications (Apps) for measuring visual acuity [37]. One study in Kenya showed that the Peek Visual Acuity App was a repeatable, accurate and reliable measure of visual acuity in adults [38]. This App was found to be acceptable to patients, care givers and stakeholders [39]. Another study among school-going children compared the performance of teachers using the Peek Acuity App to assess children’s vision to a clinician assessing the same children using as standard backlit EDTRS LogMAR visual acuity test chart found a sensitivity of 77% (95% confidence interval [CI] = 64.8–86.5) and specificity of 91% (95% CI = 89.3–92.1) [40]. We initially developed and validated the ‘Peek community screening App’ that allows referral decisions to be made precisely and reliably across all ages for the trial. Results from the validation of this App showed that community volunteers (CV) could accurately make referral decisions (manuscript in preparation).

A recent systematic review showed that mobile health (m-Health) interventions that support communication between healthcare providers and patients through short messaging service (SMS) appointment reminders are beneficial [41]. Similarly, outreach service provision in India incorporated the electronic transfer of health-related data from outreach clinics to base hospitals with some success [42]. This provides an opportunity for a combined outreach model, which incorporates triage and referrals aided by mobile technology.

We recently conducted a cluster randomised controlled trial in primary schools in Kenya using the Peek School Eye Health system. The system uses the Peek Acuity App to detect VI in school children. For those that then screen positive and who require further assessment or follow-up, it generates automated text messages to parents/guardians and contact teachers, as well as real-time notifications to hospital services. We found that teachers could reliably screen for VI. Uptake of referrals to eye care providers was substantially higher in the Peek intervention arm of this school trial [40]. This trial provided evidence that m-Health solutions could be used to improve access to eye health services.

In this new trial, the Peek Community Eye Health (Peek CEH) system will be compared to the current standard approach of periodic health centre-based outreach clinics. The system uses the ‘Peek Community Screening App’, which is a smartphone-guided algorithm for supporting ‘Peek Users’ to identify and refer people with visual impairment and other eye problems in the community. Peek Users are CVs who are trained specifically in how to use Peek. They travel to multiple communities to perform their duties. During community outreach, they work with the local CVs to identify and refer patients needing ophthalmic attention. Although treatment will be provided at no cost, it is assumed that: (1) all patients trust the health system; (2) eye health workers have the capacity and able to manage all conditions; and (3) relevant treatment modalities will be available.

### Objectives

The objective of this cluster randomised trial is to test the hypothesis that the Peek CEH system can increase access to eye services through: (1) increased identification of people with impaired vision and eye problems in the community; (2) increased uptake of a referral within four weeks by patients with identified an eye problem; and (3) more appropriate utilisation of primary and secondary care services at each health system level.

## Methodology

This protocol is structured in accordance with the Standard Protocol Items: Recommendations for Intervention Trials (SPIRIT) 2013 Checklist [43] (see Additional file 1).

### Trial design and overview

This trial is a single-masked, parallel-group, cluster randomised controlled trial. Thirty-six community units with their health facilities (dispensary or health centres) will be randomly selected to receive either the intervention (community screening using the Peek screening system) or the current standard of care (periodic health centre-based outreach clinics). The health workers involved in the study will be trained to ensure standardised screening. Participants who provide consent will be enrolled to the arm to which their cluster is randomised.

In the Peek arm, all households in the cluster will be visited in turn. Consenting individuals will have their visual acuity tested using the Peek visual acuity screening application on a smartphone. All participants with reduced visual acuity or reporting another eye problem will be referred to the linked PHC for assessment and management. Those requiring treatment not available from the PHC facility will be referred onwards to Kitale Eye Unit (KEU). In the control arm, communities will be notified about the periodic eye health outreach clinic that will be held in the local health centre. People attending this service will be assessed and, if necessary, referred onwards to KEU.

The participants will be followed up for eight weeks after referral from the community. The primary outcome will be the number of people per 10,000 population (rate) attending triage at a local health facility (PHC) with any confirmed eye conditions (true-positive cases determined at triage by hospital outreach team) following a referral or by self-referral within four weeks from the time of sensitisation. The secondary outcome will be the proportion of people referred from the PHC triage attending their referrals at KEU within four weeks of being referred. A participant (standard or Peek) who attends the hospital appointment within four weeks will be considered an ‘attender’ while anyone who is referred but does not attend within the same time is a ‘non-attender’.

### Participant timeline and study flow chart

**Fig. 1:**
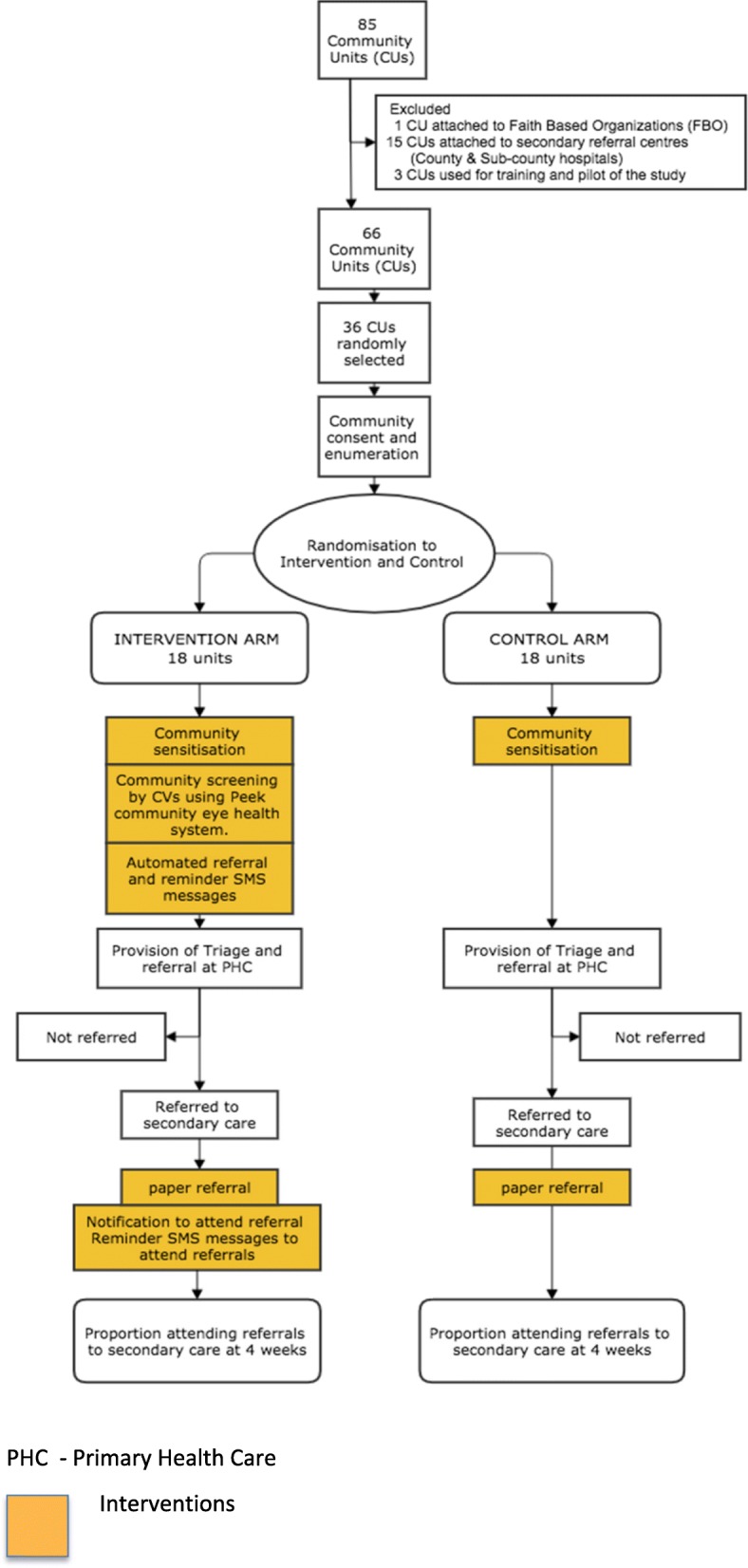
Trial design outline: randomisation, interventions and flow of participants

The study flow chart and participant timeline are presented in Fig. 1 and Table 1, respectively.

**Table 1:** Project timeline

	Study period											
	Enrolment		Allocation	Post allocation								Close-out
Week	– 2	− 1	0	1	2	3	4	5	6	7	8	9
Preparation												
Training of field workers	X	X										
Approvals: Trans Nzoia health department and head of health facilities	X											
Community enumeration and obtaining consent	X	X										
Allocation of community units			X									
Interventions												
Community sensitisation				X								
Peek package (community screening, automatic reminder short text messaging)					X	X	X					
Standard care				X								
Triage treatment camp								X				
Peek referral reminders to attend Kitale Eye Unit (automatic reminder short text messaging)								X	X	X	X	X
Assessment												
Attendance (uptake) of referrals								X	X	X	X	X

### Participants, interventions and outcomes

#### Study setting

The trial will be conducted in community units that are served by government-run dispensaries and health centres in Trans Nzoia County in northern Kenya. Trans Nzoia County has a population of 818,757 people (2009 census) of which 407,172 (49.7%) were male [44]. It is organised into five sub-counties. There were 173,719 households, with an average of five people per household. The large majority have no Internet access (669,347, 81.8%) [45]. There are 61 government facilities (six hospitals, 12 health centres, 43 dispensaries) and 76 facilities owned privately or by faith-based organisations [46]. Eye services are offered at KEU and through outreach services, provided by eye care staff from KEU to other health facilities. Screening and treatment of eye conditions (triage) is offered during outreach. The trial will be coordinated from Kitale Hospital by a team consisting of a programme manager, administrator, ophthalmic nurses, field workers and an ophthalmologist.

#### Cluster definition

**Fig. 2:**
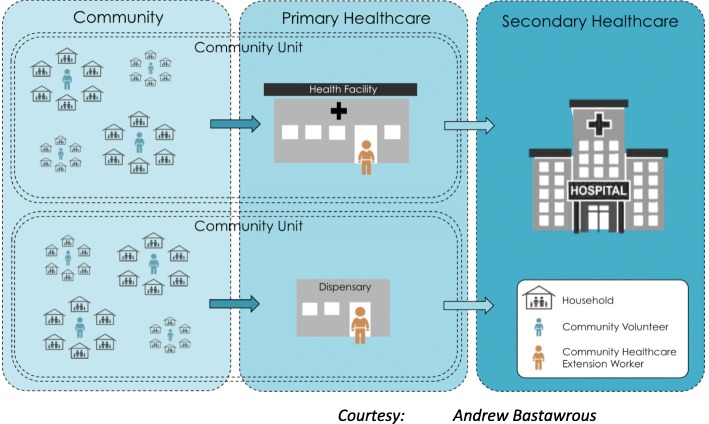
Community units, levels of healthcare and referral pathway (prepared by Andrew Bastawrous)

The unit of randomisation for this trial will be Community Units (CU). These are defined as a dispensary or health centre together with the community they serve (Fig. 2). A typical CU comprises a population of 5000–10,000 people. It has a dispensary or health centre, staffed by one or two Community Health Extension Workers (CHEWs). Associated with each CU, there are usually 20–50 CVs [47]. The CHEWs based at the health centre or dispensary train, support and supervise the CVs. To date, 85 CUs have been established and personnel trained in this county [46]. CUs were chosen because it represents the future shape of healthcare in Kenya; they are distributed throughout the county and have a good referral network that provides linkages between community and health system. The CUs with untrained personnel provide a buffer zone that will minimise contamination.

#### Cluster eligibility criteria

A list of all health facilities with their geo-coordinates as well as corresponding CUs and catchment population will be obtained from the Trans Nzoia County Department of Health. The location of each hospital will be determined using Google Maps. Health facilities without CUs, those with existing screening programs and the communities directly served by KEU will be excluded. We will also exclude all the non-government health facility-associated CUs. From the remaining 66 CUs, a total of 36 CUs will be randomly selected for the study. A restricted cluster random sampling technique (described below) will be applied to allocate the selected CUs to the Peek intervention (18 CUs) or the standard care group (18 CUs). The restriction will be based on the distance and location of the CU’s heath facility relative to KEU.

#### Participant eligibility criteria

All people who consent to participate and present in the community unit area during the study period will be included. People who are unwilling to give consent or who have had an eye condition treated at hospital within two weeks before the beginning of the study will be excluded.

#### Interventions

A comparison of the two arms is shown in Table 2 and Fig. 1. Before the beginning of the trial, households in each of the clusters in both arms will be visited by the field team to explain the study, obtain consent (see Additional file 2) and enumerate the residents. Parents/guardians will provide consent for children. At the beginning of the trial, in both arms, there will be posters and verbal notices (churches and schools) advertising the forthcoming outreach clinic for eye checks, encouraging people with eye problems to self-report to the clinic on a specific date when the team will visit.

**Table 2:** Comparison of the interventions in the two arms of the trial

	Intervention arm	Control arm
Consent and enumeration	Yes	Yes
Community sensitisation	Posters and announcement in churches and schools	Posters and announcement in churches and schools
Community screening	Vision assessed at household level using Peek E- acuity by field worker	No vision assessment at household level
	Screening decision using Peek Screening App	No screening
	Personalised text and weekly reminder messages for participants/carers in the relevant local language to attend appointments	No text message
Referral from community to PHC (triage centre)	Self-referring participants and referrals by CV using Peek system?	Self-referring participants
	Automatic referral through Peek system	No referrals
Provision of triage	Trained team composed of ophthalmic clinical officer, ophthalmic nurses and two field workers	Trained team composed of ophthalmic clinical officer, ophthalmic nurses and two field workers
Referral from triage centre to secondary care	Paper referral	Paper referral
	Automatic referral through Peek system and weekly reminder SMS	
Assessment of primary outcome	Same for both arms (trained field worker)	Same for both arms (trained field worker)
Assessment of referrals	Ophthalmic clinical officer	Ophthalmic clinical officer

##### Peek CEH intervention arm

In each cluster, a small mobile team of a ‘Peek User’ (CVs trained specifically on how to use the Peek Community Screening App and who travel to multiple communities to perform their duties) and local CV will visit each household. The CV, a person from that same community, will guide the Peek User around the village. After reconfirming consent, people who are resident in the household at the time of the visit will have a vision assessment. The visual acuity of each eye will be measured separately using the Peek Acuity App [38]. This smartphone application presents a series of E-optotypes in one of four orientations, selected at random. The test algorithm prompts the following screening questions to the parents or guardian with a child (‘*Does the child have any problem with their eyes today?*’) or directly to participant themselves (‘*Do you have any discomfort or pain in your eyes today?*’ and ‘*Do you have a problem with your sight when seeing far or near objects?*’)*.* If the participant is aged ≥ 6 years, the App prompts for distant visuals acuity assessment using Peek Acuity App and assessment of near visual acuity for all people aged ≥ 40 years. Near vision will be assessed at 40 cm using the RADNER reading chart [48]. They will be referred to the PHC for subsequent assessment by the visiting time if: the visual acuity is < 6/12 in either eye; there is any self-reported eye pain or discomfort; there is difficulty seeing distant or near objects; or they are not able see N8 on near vision assessment. Household members absent during the first visit will be asked to join the examination team at the next household or next day.

Those who have reduced visual acuity on screening or report an eye problem will be referred to a health post for triage on a specific date when the KEU team visit. The system will generate several SMS text messages: (1) to the patient and family associate asking them to present to the health facility on a specific day (set to be within four weeks); (2) the CV will receive an SMS list of patients from their community that have been referred; and (3) the CHEW responsible for that CU will similarly receive the same list of referred patients. A weekly reminder SMS will be sent to the patient for them to attend their referral appointment with the last reminder being one day before the appointment.

On the pre-advertised date, a team from KEU will be based at the CU’s dispensary. The participants referred from the household screening because of reduced vision or a specific eye problem will be reminded to attend. They will assess the presenting patients using the current standard procedure (Snellen chart visual acuity, magnifying loop, refraction and direct ophthalmoscopy when indicated). They will provide simple treatments or refer patients to KEU for further assessment as indicated. A pre-numbered paper referral letter will be given to the patient to present at KEU. The referral slip has their study number, name and triage centre, and telephone number, and indicates that assessment and treatment will be provided at no cost. It is expected that they will report to KEU within four weeks from being referred.

Immediately after referral from the PHC, an SMS will be sent to the patient and the family associate asking them to present to KEU. A weekly reminder SMS will be sent for those who have not attended their referral to KEU. An SMS with a list of patients who have not attended their referral will be sent to the CHEW responsible for the PHC.

##### Standard of care (control) arm

In the control arm, there will be no active Peek screening in the community; however, potential participants with eye problems at the community will be notified through community sensitisation (posters and local announcements) that if they have an eye problem to present themselves to the health facility for the triage clinic on a specified date. On that advertised date, the team from KEU will conduct an outreach clinic within the CU, which will be identical to the ones in the Peek arm described above. If an individual needs to be referred to KEU, they will be given an identical referral letter to the ones used in the Peek arm. Each letter will have a unique code number to link the patient referral record to their KEU attendance.

#### Outcomes

##### Primary outcome

The primary outcome is the number of people per 10,000 population (rate) attending triage at a local health facility (PHC) with any confirmed eye conditions (true positives) following a CV referral or by self-referral, within four weeks from the time of sensitisation. The rate will be based on baseline enumeration census for each CU. The true positives will be determined at triage by the hospital outreach team.

##### Secondary outcomes

The secondary otucomes are: (1) the number of people per 10,000 (rate) attending the triage post without any eye condition (false positives) as determined by the eye team; (2) the number of people per 10,000 population (rate) attending KEU within four weeks after being referred from PHC; (3) the proportion of participants referred from the PHC who attend the referral at KEU within four weeks of being referred from a PHC; and (4) the time taken by a participant referred from PHC to attend KEU.

#### Sample size

The sample size of 36 clusters was determined using the Hayes formula for rates in unmatched cluster randomised trials [49]. In Trans Nzoia County, a typical health facility has a catchment population of 5000 people [46]. During previous community outreaches to these health facilities, about 50–100 new patients attended. This translates to an average rate of 15 per 1000 population [50]. Assuming an intraclass correlation coefficient of 0.001, desired power of 90% and significance level of 5%, a sample of 36 CUs (18 in each arm) would be sufficient to detect a difference of 0.5%, from 1.5% in the control arm to 2.0% in the intervention arm (a 33% relative change) in overall attendance rates.

#### Assignment of interventions

##### Allocation

There are 66 potentially eligible CUs in the county (see above). We will select 36 CUs for inclusion in the trial. In order to ensure balance between the arms, restricted randomisation will be used. A list of the 66 CUs with their sub-county, distance from Kitale and direction from Kitale (categorised into four quadrants, North, South, East and West) will be compiled and used during randomisation. A statistician, who will not participate in recruitment, will generate a random allocation sequence. Randomisation will consider the direction, cluster size and distance from the hospital. The following restrictions will be used in the randomisation:

- each arm must include at least two CUs from each sub-county;
- each arm must include at least two CUs from each direction of North, South, East and West;
- the ratio between number of CUs in each arm from each direction must be in the range of 0.67–1.5;
- the difference in mean health centre distance from Kitale in each of the arms should not be > 4 km; and
- there should not be more than one CU per link health facility.

A list of 10,000 valid permutations will be generated and checked that there are no clear deviations in randomness (e.g. pairs of health centres that occur within the same arm considerably more/less often than would be expected by chance). One of these 10,000 permutations will be computer-selected at random. A list of CUs allocated to the control group, intervention group and those not involved will be prepared.

In health facilities where there are larger catchment populations and served by more than one CU, one of the CUs will be randomly selected along with its population unit, so that the size of the clusters studied is around 5000.

#### Masking

It will not be possible to mask the participants or the health workers from the intervention to which they are allocated; however, the study statistician, hospital registration clerk and clinician assessing outcomes will be masked. The data clerk will be masked to the intervention arm because all the patients will present with paper referral. The clinician assessing secondary outcomes will not participate in patient recruitment or assessing attendance and all patients will be given similar assessment questionnaires. The statistician will not participate in patient recruitment.

#### Data collection, management and analysis

##### Data collection

In both arms, we will use electronic data capture and management using dedicated Peek software with built-in consistency checks. In both arms, this will include the enumeration data, the triage data in the health centre/dispensary and the outcome data collected in the KEU. In addition, the household screening data will also be captured electronically for the Peek arm during the study period and in the control arm following the study when the team will screen all the control clusters. Field workers will be provided with tablets for data entry. Information will be backed up regularly.

During triage assessment at the health centre/dispensary, trained field workers will verify that the participant comes from the catchment population. From each eligible participant, date of attendance, name, age, gender and own or parents’ mobile phone number, whether referred using the Peek system or self-referral, the diagnosis and treatment plan (treated or referred) will be obtained. At KEU, all referred patient will be marked as attended upon presentation and record the date of visit, diagnosis and outcome of the visit.

#### Data management

Data will be entered directly onto smartphones by trained field workers and uploaded to a secure server once connected to the Internet before being exported into Stata for analysis. The database will be encrypted and password-protected. At the end of the study, the data will be archived at LSHTM.

##### Data analyses

The trial will be reported using the 2010 CONSORT guidelines, with the cluster RCT extension [51]. Analysis will be by intention to treat. Socio-demographic characteristics of participants at baseline will be tabulated by arm: age; sex; residence; and distance from hospitals (categorised distances). The distributions of these variables by intervention arm will be compared to assess whether there is imbalance at baseline in these potential confounding factors.

#### Analysis of the primary outcome

The proportion of individuals attending triage within each cluster will be calculated, by dividing the number attending triage and having a confirmed eye condition by the cluster population, which will be determined by the baseline enumeration census in both arms (true-positive attendance rate). A t-test will be performed on these cluster-level rates providing an estimate of the rate difference (with a 95% CI) between the two arms and a *P* value in order to assess the strength of evidence against the null hypothesis that the rate is equal in the two arms [52]. The two study arms should be balanced in terms of confounders due to the restricted randomisation process so the primary analysis will be unadjusted.

#### Analysis of secondary outcomes

The proportion attending triage but having no eye condition (false-positive attendance rate) will be estimated in a similar manner to the above in both arms, with a rate difference estimated, along with its 95% CI.

In order to estimate the effect of the intervention on the attendance rate of true positives at KEU the approach will be identical to the cluster-level analysis of the primary outcome. The numerator of each cluster is the number of individuals attending KEU following a referral from triage and the denominator is the cluster population. Again, a t-test will be used to assess the evidence as to whether the rate differs between arms and the analysis will again be unadjusted.

The difference in the proportion of patients referred from the PHC to the KEU who attend their referral within four weeks, by arm, will be tested using a random effects logistic regression, with attendance at KEU as the outcome, trial arm as the primary exposure and cluster as a random effect to account for within cluster correlation. Due to the fact that the characteristics of the patients referred in each arm may be different (due to the potential upstream impact of the intervention), this analysis will be adjusted for sex, age group and distance from KEU.

The impact of the intervention on time-to-attendance will be investigated, using Kaplan–Meier plots for each arm to compare attendance of referral. The hazard ratio will be estimated using Cox regression, again adjusted for sex and age group, to assess whether patients referred in the intervention arm attended their referrals sooner than those in the control arm..

We will assess possible effect modification of sex, age and distance from KEU. In the cluster-level analyses, the approach recommended by Cheung et al. [53], will be used for age and sex, where the rate in each group within each cluster will be estimated, then the difference in this rates in each group found, before finally performing a t-test on these differences by arm. In order to identify if the distance from KEU is an effect modifier, since it is a cluster-level covariate, this can be done by performing a linear regression on the cluster level rates and include distance and trial arm as exposures with an interaction term between them. For the individual-level analyses, an interaction term will be included with trial arm for each of the potential effect modifiers (age, sex, distance from KEU).

### Monitoring

#### Data monitoring

The study presents minimal risk and we do not anticipate significant adverse events. Therefore, a data and safety monitoring committee was not considered necessary; however, an audit will be done by the London School of Hygiene and Tropical Medicine (LSHTM), the Trial Sponsor, if it is deemed necessary. No interim analysis is planned due to the relatively short duration of the study.

#### Harm

The tests being done are in routine clinical use in Kenya and internationally. There are no anticipated harms from this non-invasive assessment process in either arm. Assessment in the community will take 5 min per person. Experienced certified ophthalmic clinical officers will provide treatment for all participants with eye problems, under the supervision of an ophthalmologist.

#### Protocol amendments

There have been no protocol amendments since the initial application. Amendments to the protocol are not currently anticipated; however, if they are required they will be submitted to the two committees mentioned above.

#### Consent

Trained field workers will obtain written informed consent from all participants. Where an individual is unable to read, the information will be read to them and their consent documented by thumbprint, in the presence of an independent witness. Consent for children will be obtained from parents or guardians accompanying them. A copy of the information sheet will be given to each participant. Verbal assent will also be obtained from children before being examined.

#### Confidentiality

Data will be anonymised before analysis and long-term storage by the removal of personal identifying information. The Peek database will be encrypted and password-protected with access only granted to staff involved in the study. Data with identifiable information will be secured within a locked project office at KEU, with limited access to only authorised staff.

#### Access to data

Investigators at LSTHM and Kitale Hospital will have access to the final trial dataset. An agreement exists on data sharing and intellectual property. All the data will be archived at LSHTM after the study is completed.

#### Post-trial care

Given that the trial is being conducted by KEU, it is integrated into existing health systems through which the patients will be managed. The control arm clusters will have the same screening service as the intervention arm after the end of the trial.

#### Dissemination

Summary of the findings will be provided for local stakeholders, Ministry of Health and participating institutions. Publications will be submitted to peer-reviewed journals (open access) and presentations made at regional and international conferences and meetings in Kenya and the United Kingdon.

## Discussion

This trial is designed to evaluate whether the Peek Screening system in the community increases access to eye services at PHC within four weeks for patients with eye problems, as well as to assess whether the same system increases uptake of referrals of people identified with eye problems from PHC to secondary care within four weeks.

One identified limitation of the study would be the number of people who will be screened and referred but have no eye problems (false positives) and may potentially overload the health system. Through the trial, we shall analyse the potential limitations with a view of understanding and providing potential solutions in the future.

The WHO and International Agency for Prevention of Blindeness (IAPB) have set a target of eliminating avoidable blindness by 2020 through early identification and treatement. This study aims to evaluate a system to reduce the prevalence of people with VI through early identification and referral from the community for those with ophthalmic ailments. The system will potentially increase access and uptake of eye services through screening and referral by CVs, for those with eye problems. Through the system, we shall be able to track the process of screening and referral of patients with a view of identifying gaps in the health system and advise policy makers on potential solutions. The results will therefore be relevant and contribute towards realising this goal.

## Trial status

At the time of submission, recruitment was ongoing. Recruitment started on 26 November 2018 and is expected to be completed on 09 April 2019. It was registered by Pan African Trials Registry on 8 June 2018.

## Additional files

Additional file 1: Standard Protocol Items: Recommendations for Intervention Trials (SPIRIT) 2013 Checklist. (DOC 122 kb)

Additional file 2: Informed consent materials. (DOCX 112 kb)

## Data Availability

Not applicable.

## Abbreviations

App: Application
CEH: Community Eye Health
CHEWs: Community health extension workers
CU: Community unit
CV: Community volunteer
IAPB: Internatinal Agency for Prevention of Blindeness
IMCI: Integrated Management of Childhood
KEU: Kitale Eye Unit
LSHTM: London School of Hygiene and Tropical Medicine
m-health: Mobile health
PACTR: Pan African Clinical Trials Registry
Peek: Portable eye examination kit
PHC: Primary healthcare
SMS: Short messaging services
SSA: Sub-Saharan Africa
VI: Visual impairment
WHO: World Health Organization
