# Supplementary material for: Peek Community Eye Health - mHealth system to increase access and efficiency of eye health services in Trans Nzoia County, Kenya: study protocol for a cluster randomised controlled trial
Source: Trials. 2019 Aug 14;20:502. doi: 10.1186/s13063-019-3615-x (PMC6694474; doi:10.1186/s13063-019-3615-x)
Supplement: Supplementary file 2 — Informed consent materials. (DOCX 112 kb) [file 13063_2019_3615_MOESM2_ESM.docx]

**Community screening and referral for eye problems using smartphone based algorithms in Trans Nzoia County, Kenya: a cluster randomised controlled trial.**

**Informed consent materials**

**Participant Information Sheet and Consent Form**

**Name of Principal Investigator(s):**

- LSHTM Principal Investigator: Prof. Matthew Burton
- Kenyan Principal Investigator: Dr Hillary Rono
- Peek Principal Investigator**:** Dr. Andrew Bastawrous

**Name of participating institutions:**

- International Centre for Eye Health, London School of Hygiene & Tropical Medicine, UK
- The Trans Nzoia county Referral Hospital, Kitale, Kenya
- Peek Vision, London, UK

**Introduction**

You are being invited to take part in a research study. Before you decide it is important for you to understand why the research is being done and what it will involve. Please take time to read or listen to the following information carefully. Talk to others about the study if you wish. Ask us if there is anything that is not clear or if you would like more information.

This form is designed to tell you everything you need to think about before you decide to consent (agree) to be in the study or not to be in the study. It is entirely your choice. If you decide to take part, you can change your mind later on and withdraw from the research study. The decision to join or not join the research study will not cause you to lose any of your usual medical benefits. You can take a copy of this consent form, to keep. Do not sign this consent form unless you have had a chance to ask questions and get answers that you understand. By signing this form you will not give up any legal rights.

**Do I have to take part in this study?**

No. You do not have to take part in this study.

**What is this study about?**

It is estimated that 253 million in the worldwide have visual impairment (cannot see well) and 36 million are blind as a result of eye disease, such as refractive error (need glasses), cataracts and eye infections. Out of 10 people who are blind, eight suffer blindness from causes that could be avoided or treated. The majority of these people live in Asia and Africa in places such as Transzoia County. Loss of sight can mean a lifetime of living with blindness, which is associated with considerable social and economic consequences.

The Ministry of Health in Kenya is working towards eliminating avoidable blindness by the year 2020, through early detection and appropriate treatment. Health workers in communities and health facilities are doing screening of communities in Transzoia County for visual problems. Those with visual problems are referred to the nearest health facility for further evaluation and treatment.

**What is the purpose of this study?**

This research seeks to determine whether assessment of people for eye problems using this mobile phone applications linked to reminder SMS leads to increasing numbers of people accessing hospital treatment for eye problems. We are trying to improve the systems used for screening people with eye problems in Kenya in future. To do this we are testing a new system for finding people with eye problems in the community and to compare this in a clinical trial to the current approach being used in Kenya.

**Whose help do we need?**

We need the help of people and communities that live in the areas of Transzoia County where the study will be run.

**What will happen if you agree to take part?**

In half of the communities we will use a smart phone application to screen for eye problems in the community. The screening process involves a community health worker assessing community members for eye problems using a mobile phone to guide in decision-making. This would be done in or near to your home. This involves you saying whether or not you can see certain letters held up at a short distance. We will then record the decisions made by the health workers. If you are found to have any eye problem, you will be referred to the nearest health facility further evaluation and treatment. At that health facility your eyes will be checked and if necessary you will be offered treatment either in the health facility or the eye hospital in Kitale. A reminder text message may be sent to you to attend your appointment. The total duration of the study will be 8 weeks from the time of initial contact.

In the other half of the communities we will advertise the availability of eye services on a specific date in your local health facility. If you attend this clinic, we will invite you to join the study. You will be examined and if necessary you will be offered treatment either in the health facility or the eye hospital in Kitale.

Then the data collected from people in the two different groups of communities will be compared to see if there are differences in the numbers of people coming forward with eye problems and accessing treatment.

**How many people are taking part in this study?**

We plan to recruit people and communities that are served by 18 health facilities to be assed using the smartphones and another 18 using the current method.

**What are the benefits of the study?**

The findings of this research will inform the Ministry of Health and partners how to plan for subsequent community screening and treatments. If you are found to have any eye problem, you will be referred to hospital for assessment and management. The treatment will be free, supported the County Government of Transzoia and partners.

**What are the risks of participating?**

The tests that we would perform to assess how well you see are safe and are in routine clinical use in Kenya and elsewhere. There are no known risks associated with this research. Assessments will take about 10 minutes to complete.

**Withdrawal from the Study**

You have the right to leave a study at any time without penalty. Your treatment, payment or enrolment in any health plans or eligibility for benefits will not be affected if you decide not to take part.

The researchers and sponsor also have the right to stop your participation in this study without your consent if, for example: They believe it is in your best interest or you were to object to any future changes that may be made in the study plan.

**New Information**

It is possible that the researchers will learn something new during the study about the risks of being in it. If this happens, they will tell you about it. Then you can decide if you want to continue to be in this study or not.

**Payment and reimbursements**

You will not be offered payment for being in this study.

**What will happen to your records we keep?**

All the information we collect will be kept confidential. It will be kept securely and only the people organizing or supervising the study and regulatory authority auditors will have access to it. They may also share portions of your medical record, with the groups named below:

- The National Bioethics Committee,
- The Institutional Review and Ethics Committee,

National privacy regulations may not apply to these groups; however, they have their own policies and guidelines to assure that all reasonable efforts will be made to keep your personal information private and confidential. A study number rather than your name will be used on study records wherever possible. Your name and other facts that might point to you will not appear when we present this study or publish its results. No information from this study will be placed into your medical record.

**What if there is a problem?**

Any complaint about the way you have been cared for during the study or any possible harm you might suffer will be addressed. Please use the addresses below to contact the study coordinators.

KITALE COUNTY HOSPITAL

EYE UNIT, BOX 98 KITALE, 30200

KENYA

**Who do I call if I have questions about the study?**

If you have Questions about the study contact Dr. Rono **(0722 6XXXXX**) and if you have Questions about your rights as a research subject: You may contact Institutional Review Ethics Committee (IREC) 053 33471 Ext.3008. IREC is a group of people that reviews studies for safety and to protect the rights of study paticipants.

**Who sponsored this study?**

The London School of Hygiene and Tropical Medicine is the sponsor of the study. The London School of Hygiene and Tropical Medicine holds insurance policies, which apply to this study. If you experience harm or injury as a result of taking part in this study, you may be eligible to claim compensation.

**Who has reviewed the study?**

This study was reviewed by the Institutional Review Ethics Committee (IREC) in Eldoret and the London School of Hygiene and Tropical Medicine Ethics Review Committee.

**Please note that:**

Your participation is voluntary. You are free to withdraw from the study at any stage.

If you choose not to participate, you will not be disadvantaged in any way. Your privacy will be protected.

**Contact Information:**

You can ring Institutional Research and Ethics committee **(**IREC) to confirm whether the Government of Kenya has approved these examinations (research). Telephone numbers: 053 33471 Ext.3008

You can contact Dr. Rono (0722 6XXXXX)

**You will be given a copy of the information sheet and a signed consent form to keep.**

**Thank you for considering taking the time to read this sheet.**

**Consent Form / Assent Form**

|  | **Please initial box** |
| --- | --- |
| 1. I confirm that I have read and understand the participant information sheet dated ......….. (version .........) for the above study. I have had the opportunity to consider the information, ask questions and have had these answered fully. |  |
| 2. I understand that my participation is voluntary and I am free to withdraw at any time, without giving any reason, without my medical care or legal rights being affected. |  |
| 3. I understand that sections of my medical notes and data collected during the study may be looked at by responsible individuals from the London School of Hygiene & Tropical Medicine, from national regulatory authorities or from this hospital, where it is relevant to my taking part in this research. I give permission for these individuals to access my records. |  |
| 4. I agree to take part in the above study. |  |
| 5. I understand that data about/from me/the participant may be shared via a public data repository or by sharing directly with other researchers, and that I will not be identifiable from this information. |  |

| Name of Participant *(printed)* |  | Signature/Thumbprint |  | Date |
| --- | --- | --- | --- | --- |
| In the case of a child, parent / guardian name *(printed)* |  | Signature/Thumbprint |  | Date |
| Name of Person taking consent |  | Signature |  | Date |
| The participant is unable to sign. As a witness, I confirm that all the information about the study was given and the participant consented to taking part. | | | | |

| Name of Impartial Witness  *(if required)* |  | Signature |  | Date |
| --- | --- | --- | --- | --- |

*1 copy for participant; 1 copy for Principal Investigator; 1 copy to be kept with hospital notes*
